# Deep-subwavelength multilayered meta-coatings for visible-infrared compatible camouflage

**DOI:** 10.1515/nanoph-2024-0029

**Published:** 2024-03-22

**Authors:** Chong Tan, Zhengji Wen, Jinguo Zhang, Dongjie Zhou, Qianli Qiu, Meikang Han, Yan Sun, Ning Dai, Jiaming Hao

**Affiliations:** State Key Laboratory of Infrared Physics, Shanghai Institute of Technical Physics, Chinese Academy of Sciences, Shanghai 200083, China; University of Chinese Academy of Sciences, No. 19A Yu Quan Road, Beijing 100049, China; Department of Materials Science & Institute of Optoelectronics, Shanghai Frontiers Science Research Base of Intelligent Optoelectronics and Perception, 12478 Fudan University, Shanghai 200433, China; Hangzhou Institute for Advanced Study, University of Chinese Academy of Sciences, Hangzhou 310024, China

**Keywords:** meta-coating, camouflage, visible-infrared compatibility, deep-subwavelength

## Abstract

Camouflage is a common technique in nature, enabling organisms to protect themselves from predators. The development of novel camouflage technologies, not only in fundamental science, but also in the fields of military and civilian applications, is of great significance. In this study, we propose a new type of deep-subwavelength four-layered meta-coating consisting of Si, Bi, Si, and Cr from top to bottom with total thickness of only ∼355 nm for visible-infrared compatible camouflage. The visible color and the infrared emission properties of the meta-coating can be independently adjusted. Colorful meta-coating for visible camouflage can be obtained by changing the thickness of top Si layer, while the selective high emissivity in non-atmospheric window for infrared camouflage remains. Due to the deep-subwavelength properties, the meta-coating shows high angle tolerance in both visible and infrared regions. The compatible camouflage capability of our proposed meta-coating in the visible-infrared region is validated under different environments. The deep-subwavelength, angular insensitivity, visible-infrared compatibility and large-area fabrication feasibility promise the meta-coating an effective solution for camouflage in various applications such as military weapons and anti-counterfeiting.

## 1 Introduction

Many animals (chameleon, squid, etc.) have developed the ability to suppress their characteristics and blend themselves into the environment after years of evolution, which are inspirational for the development of camouflage technology, but most of them only have the ability to camouflage themselves in certain specific spectral ranges [1]–[3]. Multispectral camouflage, especially visible-infrared compatible camouflage, has been drawing growing attention with the rapid development of detection technologies. To obtain both visible and infrared camouflage, it normally requires the device to satisfy the following characteristics [4–10]: (i) In visible region (390–780 nm), the presented color is similar to that of a background and further can be tuned to adapt different backgrounds; (ii) In infrared atmospheric transparency windows (ATWs), namely 3–5 μm mid-wavelength infrared (MWIR) and 8–14 μm long-wavelength infrared (LWIR) ATWs region, thermal radiation should be suppressed to against infrared detection equipment; (iii) In infrared 5–8 μm non-atmospheric transparency window (non-ATW), the device should have high emissivity to reduce the temperature by radiative cooling.

Over the past years, numerous types of artificially engineered structures, such as photonic crystals [6], [11]–[13], metamaterials [9], [10], [14]–[36], MXenes [37–42], metallic–dielectric nanostructures [43] and multilayer films [7], [44]–[53], have been proposed for wave regulations and camouflage applications. However, although great camouflage performances have been achieved through these strategies, these optical engineered structures are either hard to realize both visible and infrared camouflage simultaneously, or their preparation procedure usually requires high-precision and low-efficiency lithography fabrication techniques, hindering their practical applications.

In this paper, a new paradigm of cost-effective, lithography-free, multilayered meta-coatings (MMC) is presented and experimentally demonstrated for visible-infrared compatible camouflage. The proposed camouflage meta-coating basically consists of subwavelength four-layered dielectric-semimetal-dielectric-metal thin films. From top to bottom, the first and second layers are nanometer-thick amorphous silicon (α-Si) and bismuth (Bi) films, respectively. The third and fourth layers are composed of hundred-nanometer scale α-Si and chromium (Cr) films. Both theoretical and experimental results show that such MMCs can generate various colors independently as changing the thickness of the top highly absorbing dielectric medium and exhibits low emissivity in the MWIR and LWIR ATWs and high emissivity in the non-ATW (5–8 μm). Compared to previous works, the proposed four-layered meta-coating has advantages of simple structure, cost-efficiency, large-area fabrication, deep-subwavelength, and angular insensitivity. These advances would benefit their applications in visible-infrared compatible camouflage.

## 2 Results and discussion

**Figure 1:**
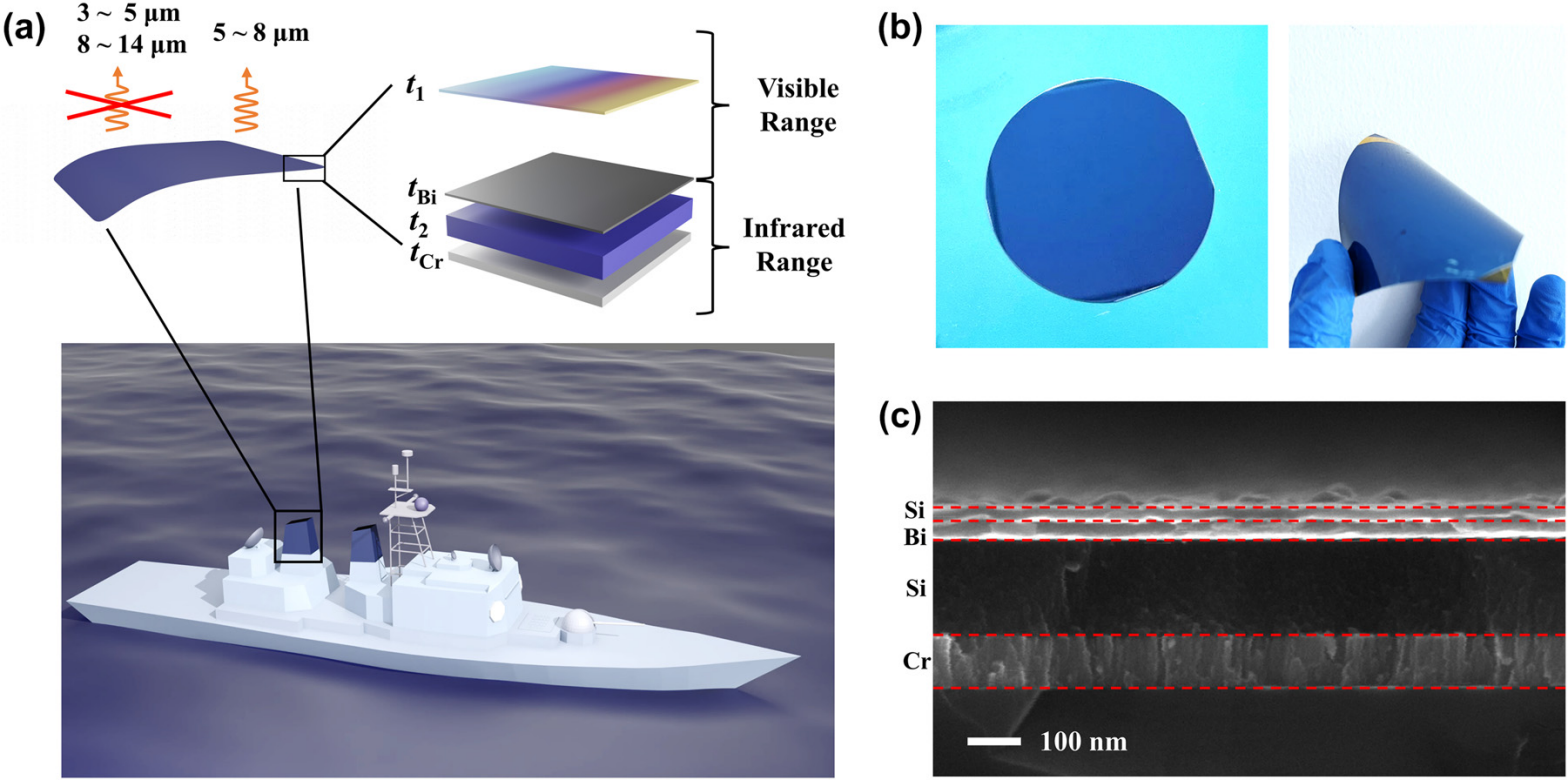
Visible-infrared compatible camouflage concept and fabrication. (a) Concept schematic of deep-subwavelength multilayered meta-coatings for visible-infrared compatible camouflage; (b) photos of fabricated samples on a 4-inch Si substrate (left panel) and flexible polyimide substrate (right panel); (c) scanning electron microscope image of part of the fabricated sample.

Figure 1(a) illustrates a schematic diagram of the proposed four-layer thin-film meta-coating. In our design, an ultrathin α-Si is selected as the top dielectric material due to its highly absorbing properties in the visible region. The second layer, Bi nanometer-thin film is adopted as the second layer, which plays an important role in such a visible-infrared compatible camouflage device. On the one hand, Bi thin film combines with the top Si layer to construct a visible subwavelength bi-layered asymmetric Fabry–Perot (FP) resonant structure for generating vivid structural colors [54]–[56]. On the other hand, it produces an infrared subwavelength tri-layered semimetal–dielectric–metal (SMDM) resonant structure along with the third and fourth layers to control infrared emission properties [57], [58]. The two types of deep-subwavelength resonances are both supported by Bi layer because of its unique optical properties (metallic properties in visible region and lossy dielectric with giant refractive index (*n* ≈ 10) in infrared region).

**Figure 2:**
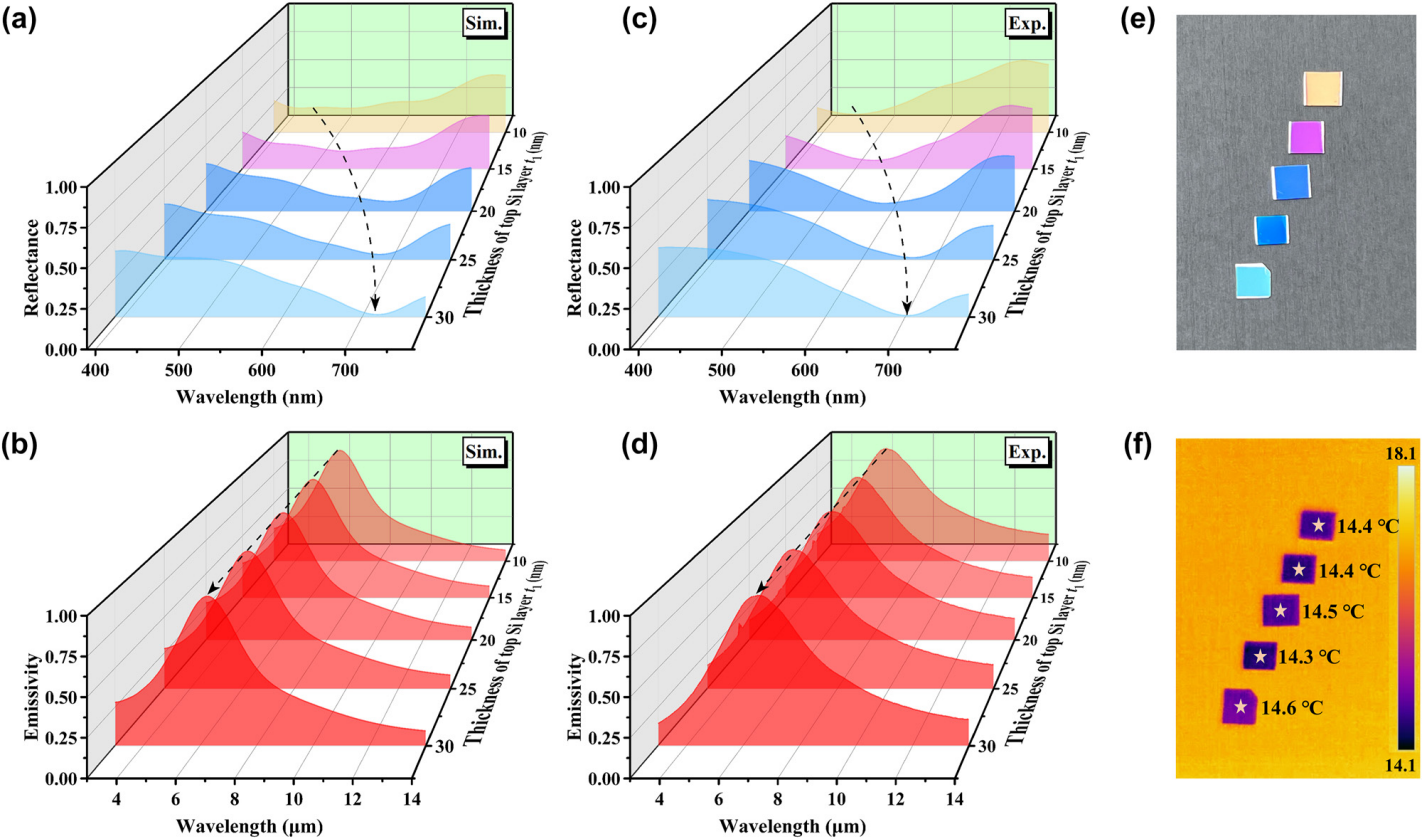
Simulated (a) visible reflectance and (b) infrared emissivity spectra of different thickness of top Si layer (*t*_1_). Measured (c) visible reflectance and (d) infrared emissivity spectra of different thickness of top Si layer (*t*_1_) (e) visible and (f) infrared images of the corresponding fabricated samples.

Numerical computations are performed to investigate the relationship between the coloration (visible reflection)/infrared emission properties and the geometric parameters of the MMC. Figure 2(a) shows the calculated reflectance spectra for five different thicknesses of top α-Si thin films (*t*_1_ = 10, 15, 20, 25, and 30 nm) in the visible range at normal incidence, while the thicknesses of other layers are taken as *t*_Bi_ = 30 nm, *t*_2_ = 200 nm, and *t*_Cr_ = 100 nm, where *t*_Bi_, *t*_2_, and *t*_Cr_ represent the thicknesses of the second Bi thin film, the third α-Si layer and the fourth Cr film, respectively. The proposed structure clearly exhibits a reflection dip, particularly, as *t*_1_ increases, the dip position becomes redshift obviously. This result indicates that the designed structures are capable of presenting different colors by the control of the reflection in the visible spectrum via the thickness adjustment of the top α-Si thin film.

Figure 2(b) displays the calculated infrared emissivity spectra of the corresponding series of structures at normal incidence. Since the emissive property obeys Kirchhoff’s law of thermal radiation, that is, under thermal equilibrium the emission and absorption of the structures are equal. The emissivity (*E*) can be obtained by *E* = *A* = 1 − *R* − *T*, where *A*, *R*, and *T* denote the absorptance, reflectance and transmittance of the proposed MMC, respectively. Regardless of the thickness of the top α-Si thin film, these structures have the same selective thermal radiation characteristics, namely, possessing low emissivity in the MWIR and LWIR ATWs and high emissivity in infrared 5–8 μm non-ATW (Figure 2(b)). This design is quite meeting the requirement of infrared camouflage as described above. From Figure 2(a) and (b), it notes that the visible and infrared properties of the designed MMC can be adjusted independently, displaying a good visible–infrared compatibility.

To experimentally demonstrate our idea, the proposed structures are fabricated using electron beam evaporation by layer-by-layer deposition (see Experimental section for fabrication details). The measured visible reflectance and infrared emissivity spectra of five fabricated coatings with the same geometric parameters as shown in Figure 2(a) are, respectively, plotted in Figure 2(c) and (d). It suggests that the experimental spectra are in good consistent with the theoretical calculations in all the cases. The fabricated prototype devices not only exhibit obvious difference in reflectance in visible range, but also have uniform low emissivity in the two infrared ATWs (*E*_3–5 µm_ = 0.39 and *E*_8–14 µm_ = 0.36) and high emissivity in non-ATW (*E*_5–8 µm_ = 0.79). The photograph of these five fabricated coatings shows that a spectrum of colors is obtained, including orange, magenta, blue, navy blue, and celeste (Figure 2(e)). A series of samples by gradually changing the thickness of α-Si layer from 10 nm to 60 nm are fabricated, and the colors based on the measured reflectance spectra of the samples are mapped in the Commission internationale de l’éclairage (CIE) 1931 color space. A wide gamut of colors from violet to red is really demonstrated, showing the potential for the visible camouflage in different background (see Figure S1 of the Supplementary Material for more details). Figure 2(f) shows the infrared image of the five samples taken by a commercial thermal infrared camera (wavelength range from 7.5 μm to 14 μm) at room temperature. Although the samples present distinctly different colors in visible spectrum, no significant difference can be seen from the infrared images since they have the same infrared emission properties.

**Figure 3:**
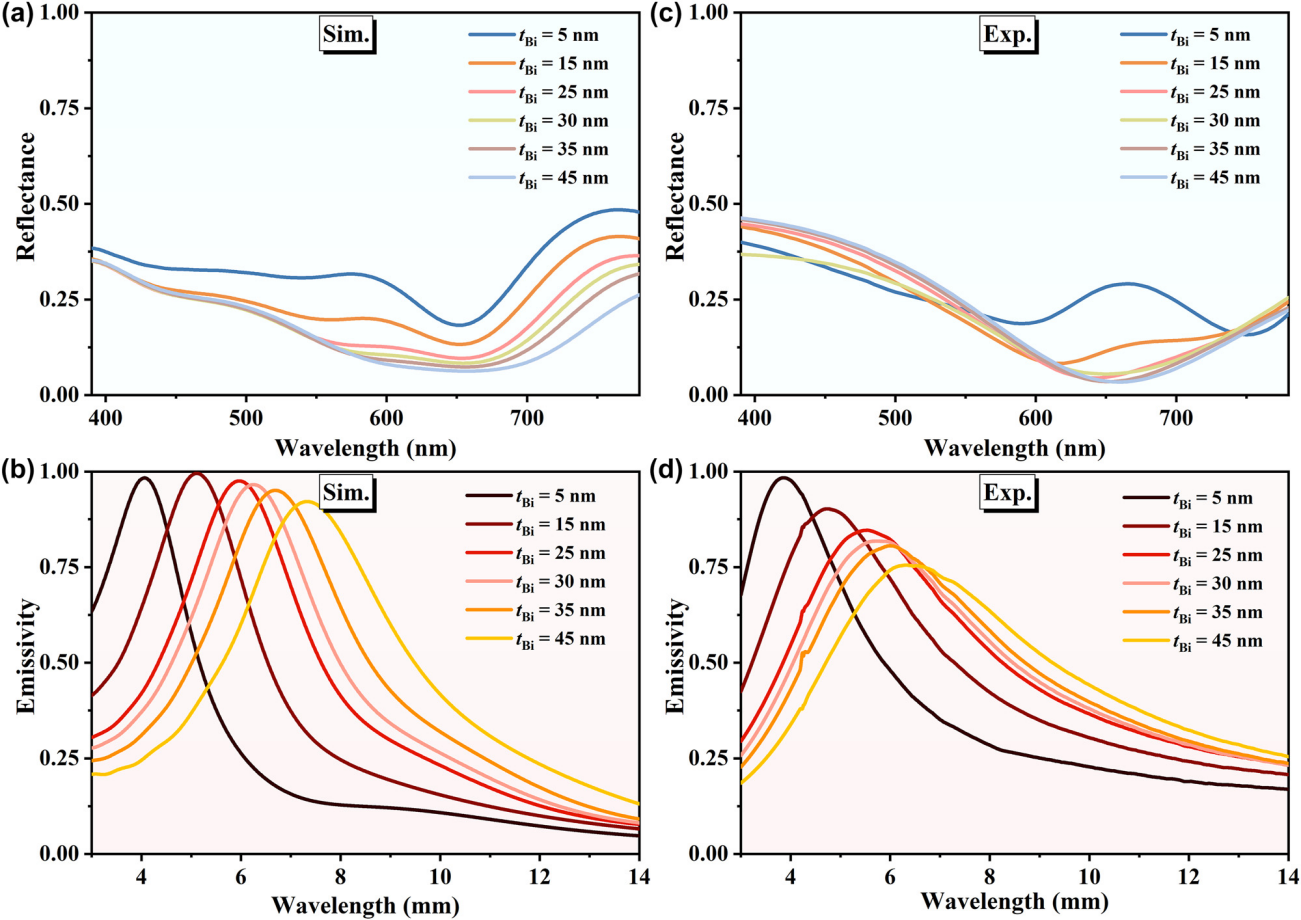
Simulated (a) visible reflectance and (b) infrared emissivity spectra with different thickness of Bi layer (*t*_Bi_). The corresponding measured (c) visible and (d) infrared spectra.

The influence of the thickness of the second layer (Bi film), on the optical properties of the MMC is further investigated. Figure 3(a) and (b) show the calculated visible reflectance and infrared emissivity spectra for a series of different thickness Bi films (*t*_Bi_ = 5, 15, 25, 30, 35, and 45 nm), with other parameters *t*_1_ = 25 nm, *t*_2_ = 200 nm, and *t*_Cr_ = 100 nm. From both visible and infrared results, there is an identical critical Bi film thickness *t*_Bi_ = 30 nm. For visible light, as *t*_Bi_ increases, the reflectance decreases gradually, until the thickness of 30 nm, it approaches zero at the wavelength of 678 nm, and much thicker Bi film has little effect on the visible reflection. For infrared light, the critical thickness corresponds to a maximum emissivity of the operating wavelength (∼6.5 μm) at the center of the non-ATW. Whereas for the case with thickness less than or greater than the critical value, the emissivity becomes smaller, and the peak operating wavelength of the emissivity will deviate from the center of the non-ATW. In order to verify the theoretical calculations, relevant experiments are performed and the corresponding measured results are presented in Figure 3(c) and (d). The comparison between the calculated and measured results shows a reasonable agreement. We infer that the discrepancies between these two results mainly originate from the imperfections of the fabricated multilayered samples, including non-uniform thickness, random fluctuation surface, and the diffusive intermixing at the interfaces.

The impact of the thickness of the third layer on the optical properties of the multilayered camouflage coating is also investigated and presented in Figure S2 in the Supplementary Material. As expected, for the case with optimal geometric parameters of other layers, the variation of the thickness of the third layer has little impact on the visible reflection property (coloration), while it will significantly change the peak wavelength of thermal emission, and even affect the peak value of emissivity for infrared light. The role of the fourth layer serves as a metallic reflector, which only needs to be thick enough to prevent light transmission.

Additionally, it is worth mentioning that our proposed camouflage meta-coatings can be deposited not only on rigid substrates such as glass and silicon wafers, but also on thin flexible substrates, as shown in Figure 1(b). Figure 1(c) shows a cross section view scanning electron microscopy (SEM) image of a fabricated sample with geometric parameters *t*_1_ = 25 nm, *t*_Bi_ = 30 nm, *t*_2_ = 200 nm, and *t*_Cr_ = 100 nm. It is obvious that the prototype device has remarkable deep-subwavelength characteristic. The top two layers that dominate the visible spectrum of the structure are 55 nm thick, much smaller than the wavelength of visible light. Even the total thickness of the sample is only 355 nm, which is also much shorter compared with the wavelength of infrared light we concerned. These results demonstrate that our proposed MMC has the advantages of low cost, good flexibility, and large-area preparation and would have broad application prospects in visible-infrared camouflage technologies.

**Figure 4:**
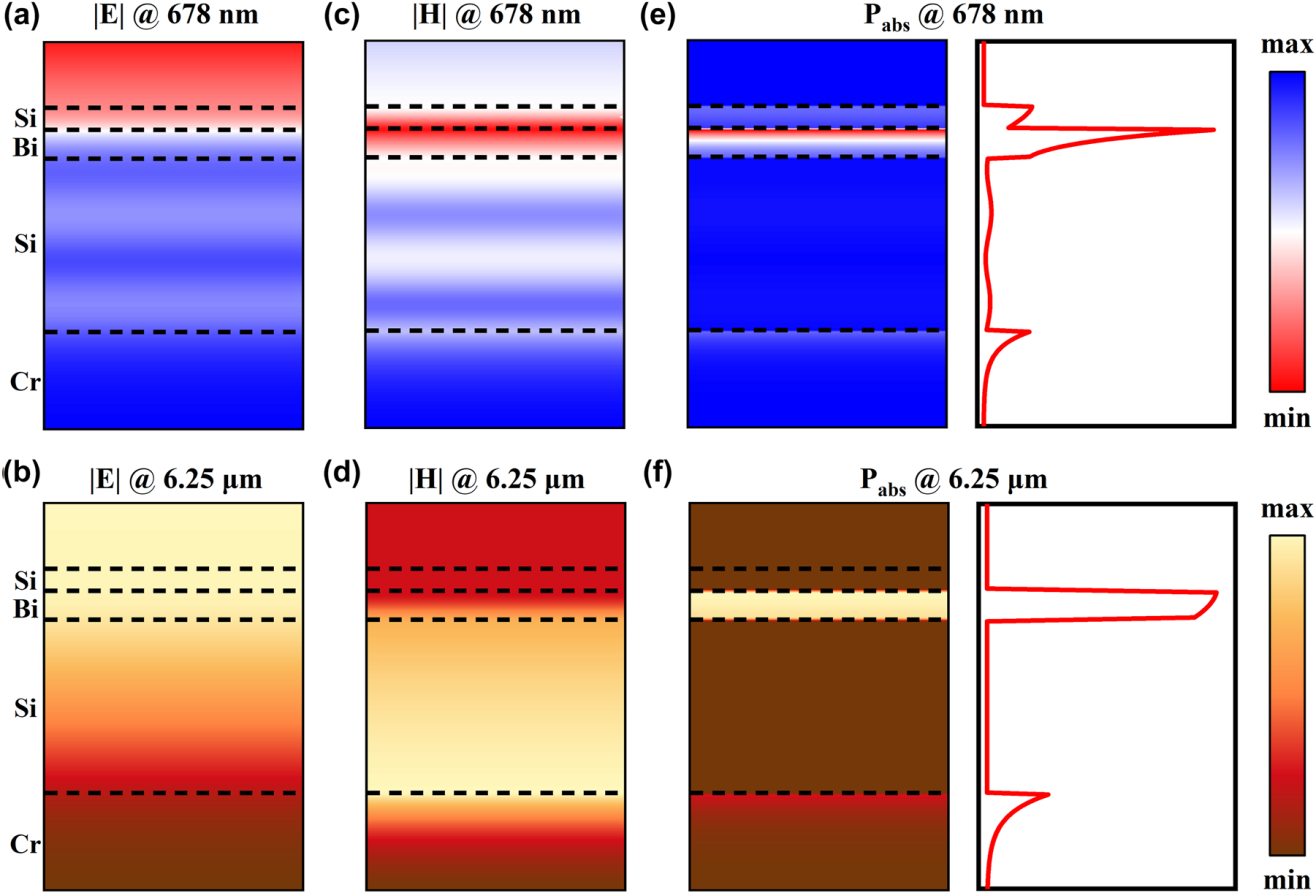
Simulated electomagnetic field and power absorption distributions. (a, b) Electric fields, (c, d) magnetic fields and (e, f) absorbed power distributions at 678 nm and 6.25 μm, respectively. The corresponding geometric parameters are *t*_1_ = 25 nm, *t*_Bi_ = 30 nm, *t*_2_ = 200 nm, and *t*_Cr_ = 100 nm.

To illustrate the working mechanism of the MMC, the distributions of electric field and dissipative power density inside the structure are investigated, as it is excited by a normally incident plane wave. Figure 4(a) and (b) show the simulated electric field and power loss profiles at the resonant wavelengths of 678 nm and 6.25 μm, respectively, where the structure has the geometric parameters of *t*_1_ = 25 nm, *t*_Bi_ = 30 nm, *t*_2_ = 200 nm, and *t*_Cr_ = 100 nm. At the wavelength of 678 nm, the most energy of incident light is localized and absorbed by the top two, α-Si and Bi, thin-film layers (Figure 4(a)), and only a small fraction energy propagates through the top two layers and inside and dissipated by the bottom two layers. In contrast, at the wavelength of 6.25 μm, the electromagnetic field is mainly distributed inside the bottom three, Bi, α-Si and Cr, thin-film layers, but the most of infrared light is dissipated by the Bi and Cr layers. Namely, the bottom three layers dominate the optical properties in infrared region. All these results firmly confirm that the coloration and infrared emission properties of the MMC are primarily determined by the bi-layered asymmetric FP resonant structure composed of the top two layers and the tri-layered SMDM resonant structure composed of the bottom three layers, respectively.

**Figure 5:**
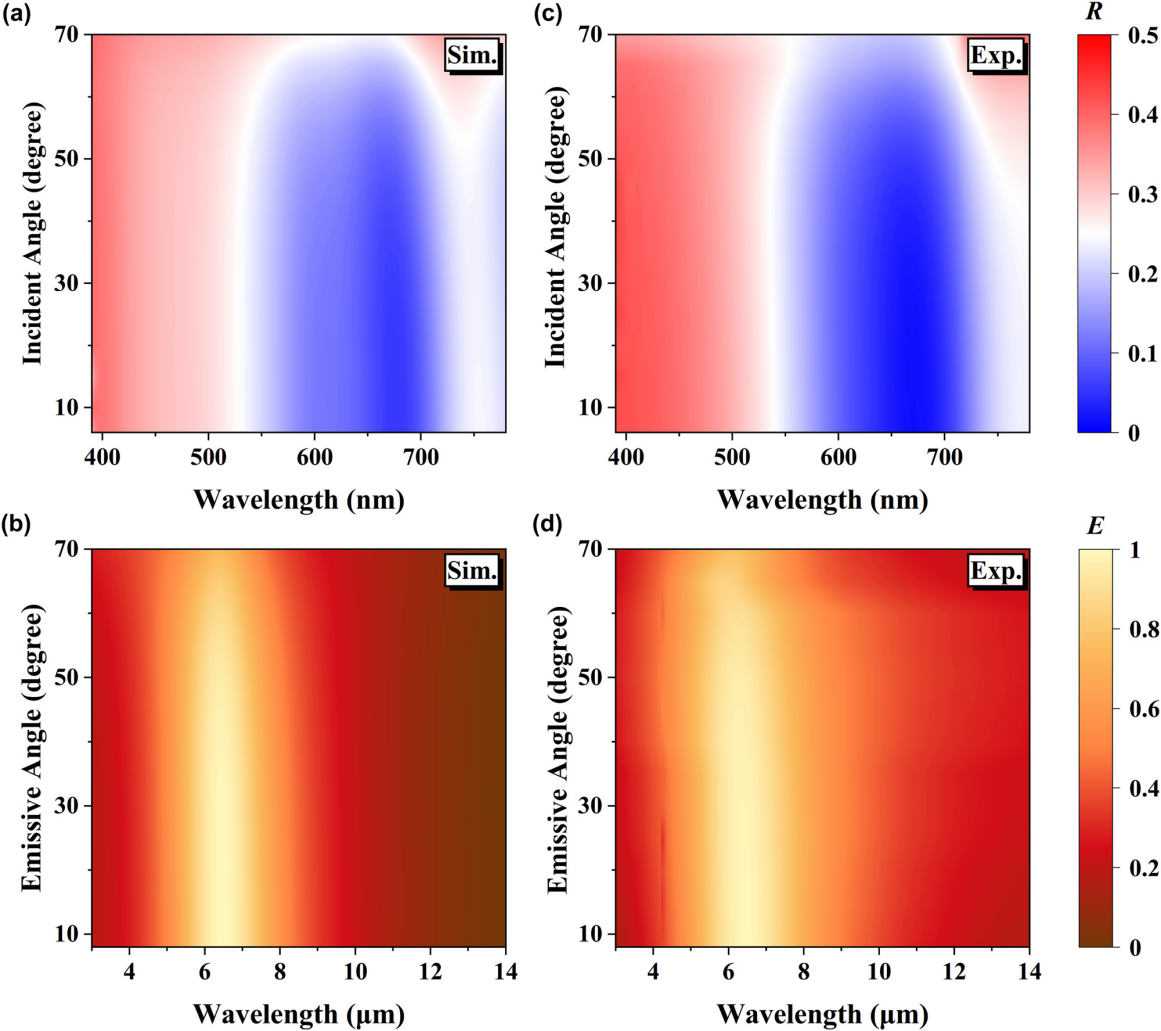
Angular dependences of optical properties of the proposed camouflage coating. (a) Simulated and (c) measured visible reflectance spectra versus different incident angles. (b) Simulated and (d) measured infrared emissivity spectra versus different emissive angles.

The angular dependences of coloration and infrared emission properties of the proposed camouflage coating are further investigated. Figure 5(a) and (b) show the calculated visible reflectance and infrared emissivity as a function of wavelength and incident (emissive) angle. Corresponding experimental results are shown in Figure 5(c) and (d). Both theoretical and experimental results indicate that the visible reflectance of the camouflage coating is almost independent of the incident angle up to 70°. Consequently, the perceptive color does not change significantly against the variation of the viewing angle (Figure S3). The angle-insensitive color effect is attributed to the strong optical asymmetric FP-type thin-film interference effects, as the thickness of the visible functional layers is much smaller than the working wavelengths, even at higher oblique angles of incidence, the propagation phase accumulation is almost negligible. Infrared emissivity spectra show a similar angle-independent effect (Figure 5(b) and (d)). This phenomenon is also ascribed to the fact that the proposed camouflage coating has the characteristic of deep-subwavelength in the infrared.

**Figure 6:**
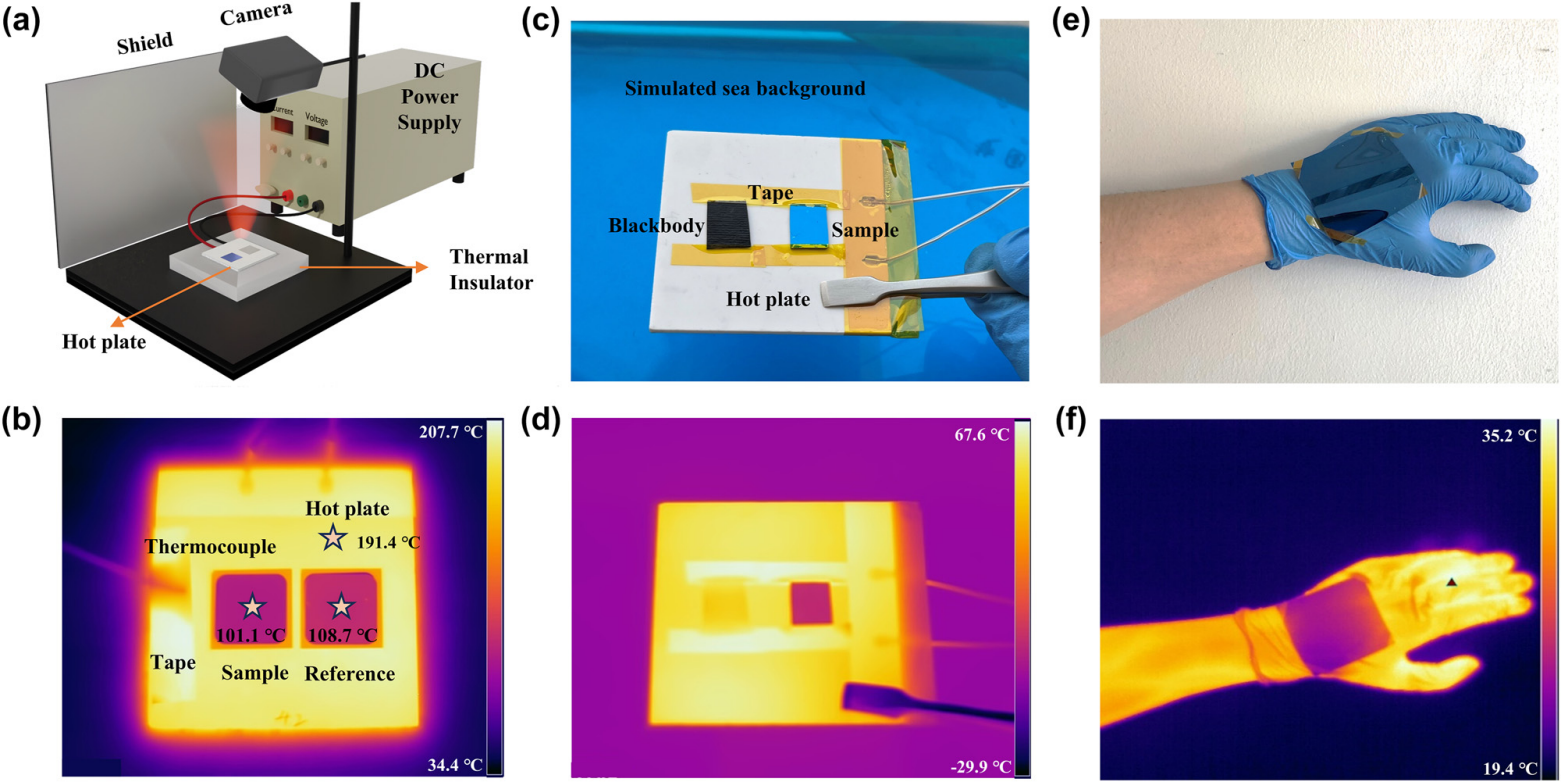
Visible-infrared compatible camouflage performance. (a) Schematics of the experimental setup for infrared image measurement of sample and reference sample. (b) The infrared image of sample and reference sample on a hot plate. (c) Digital photo and (d) infrared image of a blackbody and our sample with a blue water background. (e) Digital photo and (f) infrared image of flexible sample on a human hand.

To demonstrate the performance of the proposed MMCs, camouflage experiments of the fabricated samples are carried out under different environments. In the first case, a thermal emitter that has the same average emissivity in LWIR ATW as the MMCs but much lower emissivity in non-ATW is fabricated as a reference for comparison (the details of the reference thermal emitter are presented in Figure S4 in the Supplementary Material). Figure 6(a) shows a schematic of the experimental setup for thermal measurement in practical environment. An MMC and the reference thermal emitter are mounted on a hot plate that is powered by a DC power supply and attached by a thermocouple to record the temperature. Figure 6(b) displays an infrared image of the case taken by the thermal infrared camera, where the emissivity of the camera is set as the average emissivity at 110 °C in LWIR ATW (*E*_8–14_ _µm_ = 0.39) of the meta-coating and reference sample. The image shows that the radiation temperature of our meta-coating is 101.1 °C, which is 7.6 °C lower than the one (108.7 °C) of the reference sample. The primary origin of this temperature difference is exactly due to the difference in emissivity of the non-ATW between the multilayered meta-coating and the reference thermal emitter [44]. To verify the experimental observations, numerical computations for this case are performed, showing that the difference in the radiation temperature between the two samples reaches 7.2 °C, which is consistent with the experimental value. More importantly, theoretical analyses not only corroborate the experimental observations, but also further confirm that the lower radiation temperature of the meta-coating compared with the reference sample is really attributed to the higher thermal radiation efficiency of the former in the non-ATW than the latter (details of the numerical computations in Section S5). In the second scenario, a “blackbody” replaced of the reference thermal emitter along with a “navy blue” MMC sample are mounted on a hot plate, and the experimental setup is arranged on the top of water, as shown in Figure 6(c). Figure 6(d) displays an infrared image of this scenario captured by the thermal infrared camera (Section S6 and Figure S5). In sharp contrast to the blackbody, whether visible or infrared, the navy blue MMC sample exhibits almost identical characteristics to the background, achieving visible–infrared compatible camouflage. In the last application scenario, a flexible MMC sample is attached on the back of the hand (Figure 6(e)). The radiation temperature of the flexible MMC sample is much lower than that of the background (Figure 6(f)). It is well demonstrated by these experiments that the proposed MMC with good functionality and practicability provides an alternative solution for visible-infrared compatible camouflage under the various environments.

## 3 Conclusions

In conclusion, we have successfully developed and fabricated a large-area, flexible, deep-subwavelength meta-coating for visible-infrared compatible camouflage applications. In the visible region, the color of the meta-coating is tunable by varying the thickness of the top Si layer, enabling the coating to blend effectively with different backgrounds for visible camouflage capabilities. In the infrared region, the coating acts as a selective emitter, displaying high emissivity within the non-ATW and low emissivity within the ATWs, and the emission properties are nearly independent of the thickness of top Si layer, which means the coloration and the emission properties of our sample can be adjusted independently. These unique properties are attributed to Bi film layer which provides lossy dielectric properties in the infrared region and metallic properties in the visible region [59]. Thanks to the deep-subwavelength characteristics, the optical properties of the meta-coating are almost independent of incident/emissive angle. Additionally, we have performed experiments to assess the performance of the meta-coating in various camouflage scenarios with an infrared camera. These results demonstrate that our meta-coating can simultaneously achieve high camouflage performance in both visible and infrared regions. The finding of our study presents a straightforward approach to simultaneously and independently controlling color and thermal radiation through appropriate structural design, benefiting visible-infrared compatible camouflage applications.

## 4 Experimental section

### 4.1 Numerical computations and simulations

The numerical computations were carried out through transfer matrix methods (TMM). Electromagnetic wave numerical simulations were conducted based on finite-difference-time-domain (FDTD) method. In the simulations, a plane source was launched into a two-dimensional FDTD simulation zone. Periodic boundary conditions were imposed on the *x* axes, and perfect matched layer (PML) was imposed on the *y* axes. In visible region, the optical constants of Bi, Si, and Cr were measured and fitted by VIS-NIR spectroscopic ellipsometer (Eoptics). In infrared region, the optical constants of Bi was measured and fitted by infrared spectroscopic ellipsometer (Sendira, Sentech), and the optical constants of Si and Cr were obtained from literature [60], [61].

### 4.2 Sample fabrication and characterization

The MMC samples and reference sample were fabricated by electron-beam evaporation (Syskey Technology UHEB-LC6-03) based on layer-by-layer deposition process. The Si substrates were first taken an ultrasonic cleaning by acetone and isopropanol. The flexible PI substrates were cleaned by deionized water. The Cr, Si, and Bi layers were deposited at room temperature at rates of 0.1 nm/s, 0.2 nm/s and 0.1 nm/s, respectively. The deposition rates were monitored by gold coated crystals (Inficon) and the chamber pressure were maintained at ∼10^−6^ Torr (Pfeiffer) during the deposition process. The cross-section of the sample was characterized by scanning electron microscopy (FEI Sirion 200).

### 4.3 Spectral measurement

The infrared spectrum was measured by a Fourier transform infrared spectrometer (Thermo Scientific Nicolet iS50) over a spectral range of 3–14 µm. The visible spectrum of sample was measured by a UV–VIS–NIR spectrometer (Agilent Technologies, carry 7000 UMS).

### 4.4 Infrared image measurement

For the measurement of Figure 6(a) and (b), a commercially available silica aerogel blanket was used as a thermal insulator to reduce downward heat conduction. A hot plate biased by a DC power supply was placed on the blanket to heat the sample at about 110 °C, which measured by a thermocouple. The selective sample and reference sample were both placed on the hot plate. The infrared camera was fixed on a holder and the images were captured under the condition of stable temperature. For the measurement of Figure 6(c) and (d), the blackbody and sample are heated at about 70 °C above the simulated sea water (∼25 °C) with a depth of 1.5 cm which was distilled water with several blue dyes. For the measurement of Figure 6(e) and (f), a flexible sample was pasted on human hand by a tape.

## Supplementary Material

Supplementary Material Details
