# Supplementary material for: Deep-subwavelength multilayered meta-coatings for visible-infrared compatible camouflage
Source: Nanophotonics. 2024 Mar 22;13(13):2391–400. doi: 10.1515/nanoph-2024-0029 (PMC11501840; doi:10.1515/nanoph-2024-0029)
Supplement: Supplementary file 1 — Supplementary Material Details [file j_nanoph-2024-0029_suppl_001.docx]

**Supporting Information**

**Deep-subwavelength multilayered meta-coatings for visible-infrared compatible camouflage**

Chong Tan^1, 2, 5^, Zhengji Wen^1, 5^, Jinguo Zhang^1, 3^, Dongjie Zhou^1, 2^, Qianli Qiu^1, 2^, Meikang Han^3^, Yan Sun^1^, Ning Dai^1, 4^, and Jiaming Hao^3, *^

^1^ State Key Laboratory of Infrared Physics, Shanghai Institute of Technical Physics, Chinese Academy of Science, Shanghai 200083, China

^2^ University of Chinese Academy of Sciences, No. 19A Yu Quan Road, Beijing 100049, China

^3^ Department of Materials Science & Institute of Optoelectronics, Shanghai Frontiers Science Research Base of Intelligent Optoelectronics and Perception, Fudan University, Shanghai 200433, China

^4^ Hangzhou Institute for Advanced Study, University of Chinese Academy of Sciences, Hangzhou 310024, China

^5^ These authors contributed equally to this work

*Corresponding author: [jmhao@fudan.edu.cn](mailto:jmhao@fudan.edu.cn)

**Section S1 Color gamut characterization by adjusting thickness of top Si layer.**

**Section S2 Impact of third Si layer on optical properties.**

**Section S3 The angle-insensitive color effect characterization.**

**Section S4 Average emissivity calculation and reference sample design.**

**Section S5 Numerical calculation of temperature difference between sample and reference sample.**

**Section S6 Emissivity measurement of tap, blackbody, water, and water with blue dye.**

**Section S1 Color gamut characterization by adjusting thickness of top Si layer.**

To investigate the varies colors of sample by changing the thickness of top Si layer, we fabricate samples with thicknesses of top Si from 10 nm to 60 nm. The corresponding visible spectra at 6 degrees of our fabricated samples are shown in Figure S1(a) which are used to calculate the coordinates on CIE 1931 (Figure S1(b)).


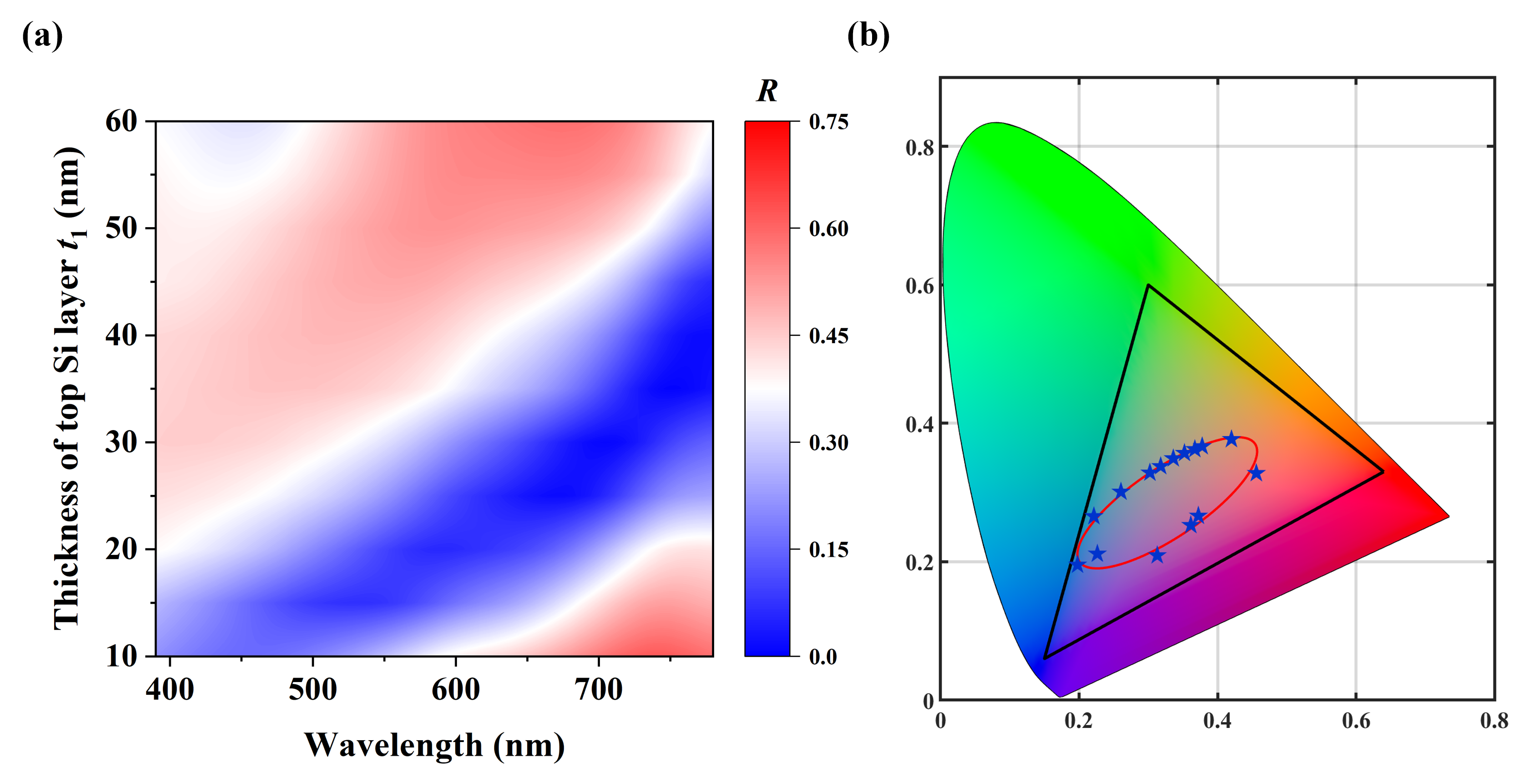


**Figure S1** (a) The measured spectra with incident angle at 6 degrees. (b) The coordinates calculated by measured spectra plotted on CIE 1931.

**Section S2 Impact of third Si layer on optical properties.**

The impact of the third Si layer on the optical properties of our meta-coatings are investigated by both numerical calculation (Figure S2(a)(b)) and experiments (Figure S2(c)(d)). The calculation and experiments are both carried out at incident angle of 30 degrees for consistency. The calculated and measured results show reasonable consistency.


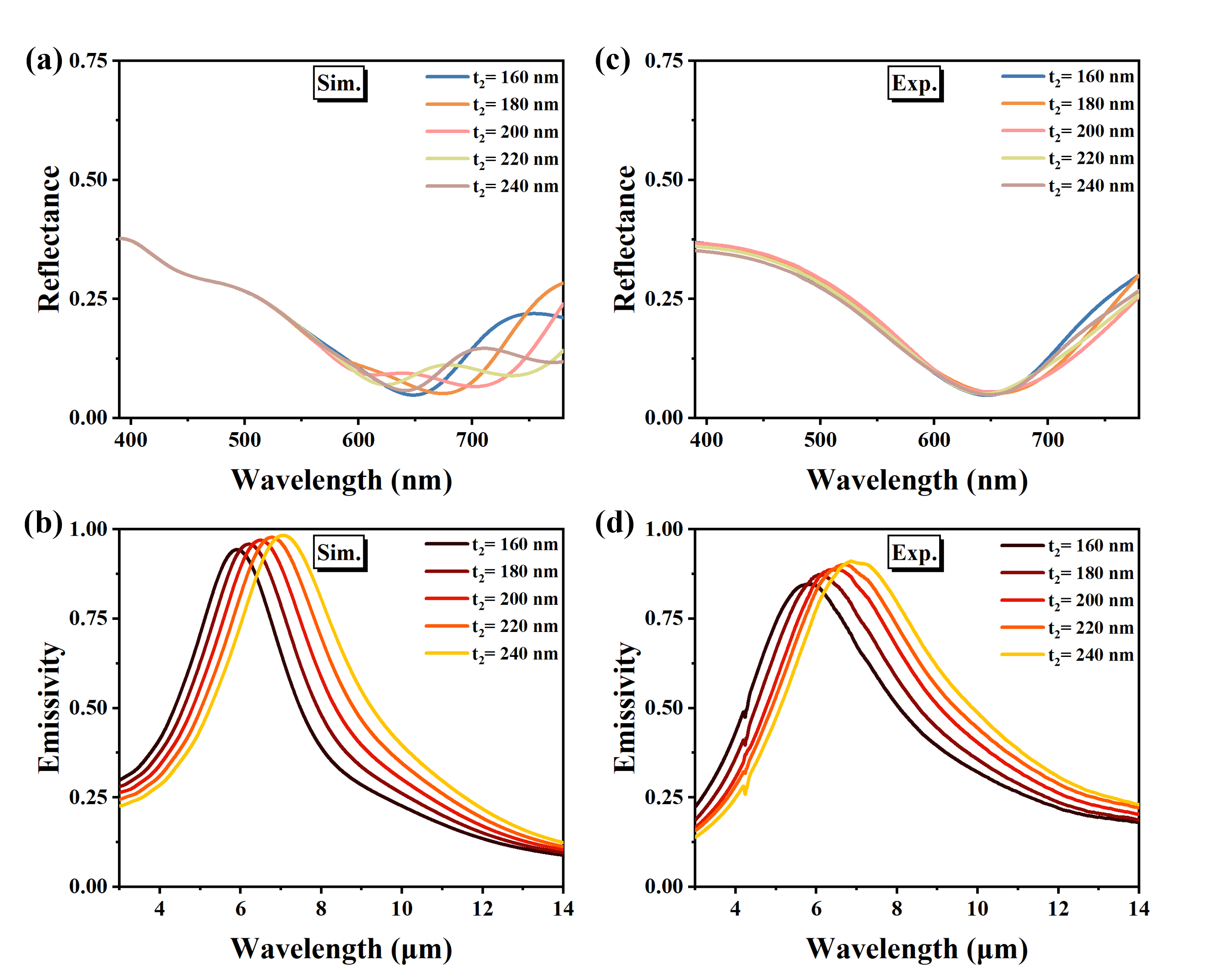


**Figure S2** Simulated (a) visible and (b) infrared spectra with different third Si layer (*t*_3_), respectively. The corresponding measured (c) visible and (d) infrared spectra.

**Section S3 The angle-insensitive color effect characterization.**

To verify the high angle tolerance of our samples for visible camouflage, we take photos of all the samples (thickness of top Si layer from 10 to 60 nm) at ~ 30 degrees and ~ 60 degrees as shown in Figure S3(a). According to the experimental measured spectra in Figure 5(c), the coordinates of different incident angle are calculated and plotted in CIE 1931 color space shown in Figure S3(b). These results show a slightly color shift of our sample under large incident angle, indicating the angle insensitive properties of our samples.


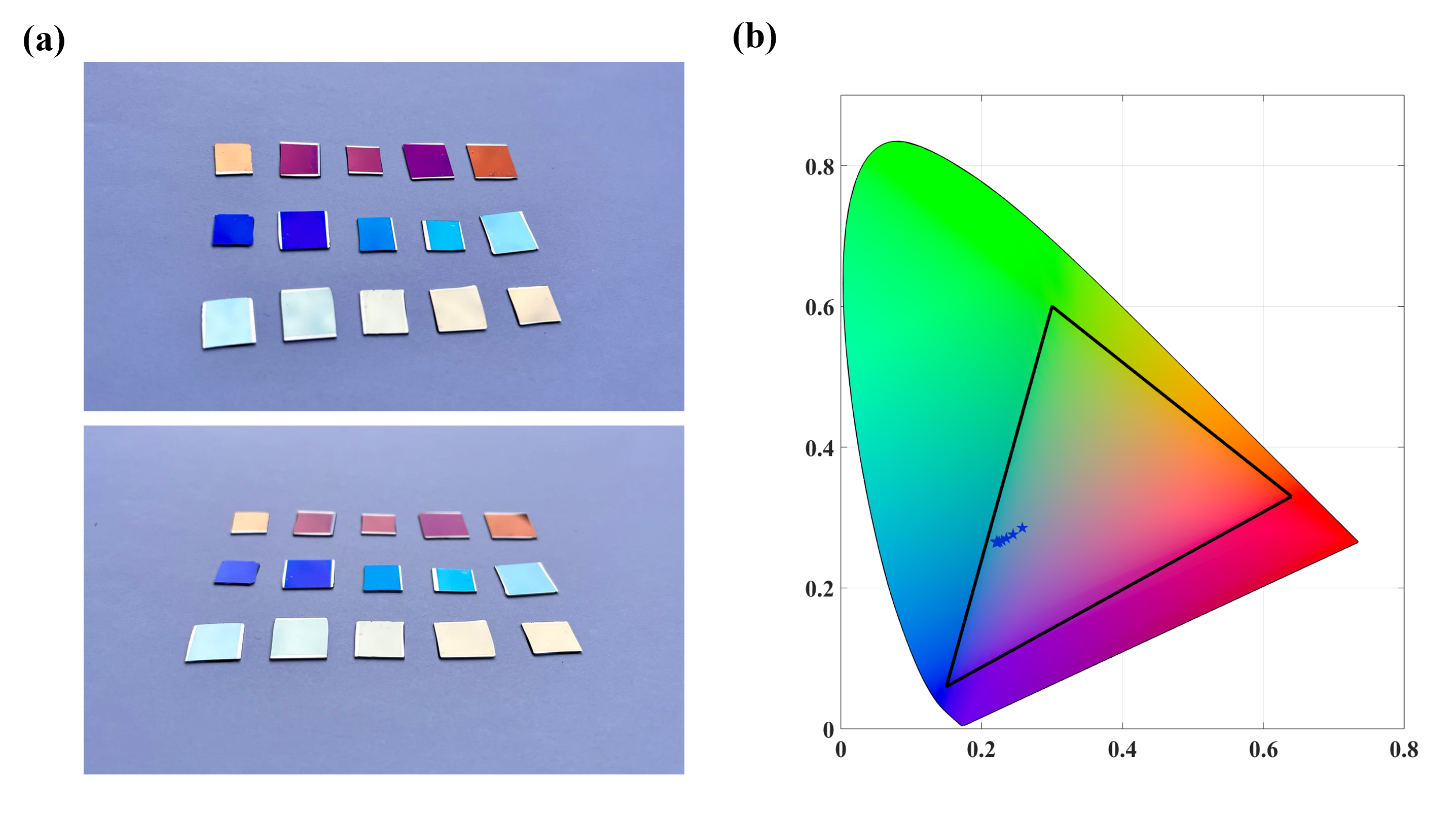


**Figure S3** (a) Visible photos taken at ~ 30 degrees (top) and ~ 60 degrees (bottom). (b) Coordinates slightly shift with increased incident angle in CIE 1931 color space.

**Section S4 Average emissivity calculation and reference sample design.**

The reference sample designed here is also a multilayer structure composed of Cr, Si and Bi. The average emissivity from $\lambda_{1}$ to $\lambda_{2}$ can be calculated as below:

$$\bar{\varepsilon}_{\lambda_{1}-\lambda_{2}}=\frac{\int_{\lambda_{1}}^{\lambda_{2}} \varepsilon(\lambda)\cdot M\left( \lambda, T \right) d\lambda}{\int_{\lambda_{1}}^{\lambda_{2}} M\left( \lambda, T \right) d\lambda}$$

where $\varepsilon(\lambda)$ is the emissivity of a sample and can be obtained by measured spectra, $M\left( \lambda, T \right)$ is blackbody radiation expressed by Plank’s Law. Thus, the average emissivity depends on the temperature. At about 110 ℃ in Figure 6(b), the average emissivity of sample calculated by measured spectrum are 0.39 (3 ~ 5 µm), 0.79 (5 ~ 8 µm), and 0.36 (8 ~ 14 µm). For reference sample, the average emissivity at ~110 ℃ in LWIR (working waveband of our infrared camera) should be as the same as the sample. Based on the designed bottom three layer, simulations are carried out for increasing the thickness of Bi and middle Si layer. Thus, the emissive peak redshift out of the LWIR, and the average emissivity become lower. By sweeping the thickness, the average emissivity of each simulated spectra is calculated until it is the same as our sample and thus the parameters are determined. Then, we design reference sample which consist of Si (10 nm), Bi (130 nm), Si (500 nm) and Cr (100 nm) from top to bottom. During the fabrication process, several reference samples are fabricated with different thickness Bi layer near the simulated parameters. Finally, spectra of reference samples are measured and the average emissivity of each sample are calculated and then choose the appropriate reference sample. The average emissivity of reference sample calculated by measure spectrum of which are 0.23 (3 ~ 5 µm), 0.16 (5 ~ 8 µm), and 0.4 (8 ~ 14 µm). For infrared image measurement, the infrared camera is sensitive in 7.5 ~ 14 µm. In this waveband, the emissivity of the sample and reference sample exhibit almost equal emissivity, which are 0.39 and 0.38, respectively. The corresponding spectra measured before and after heating on hot plate are shown in Figure S4, which indicate the thermal stability of our samples is up to 110 °C.


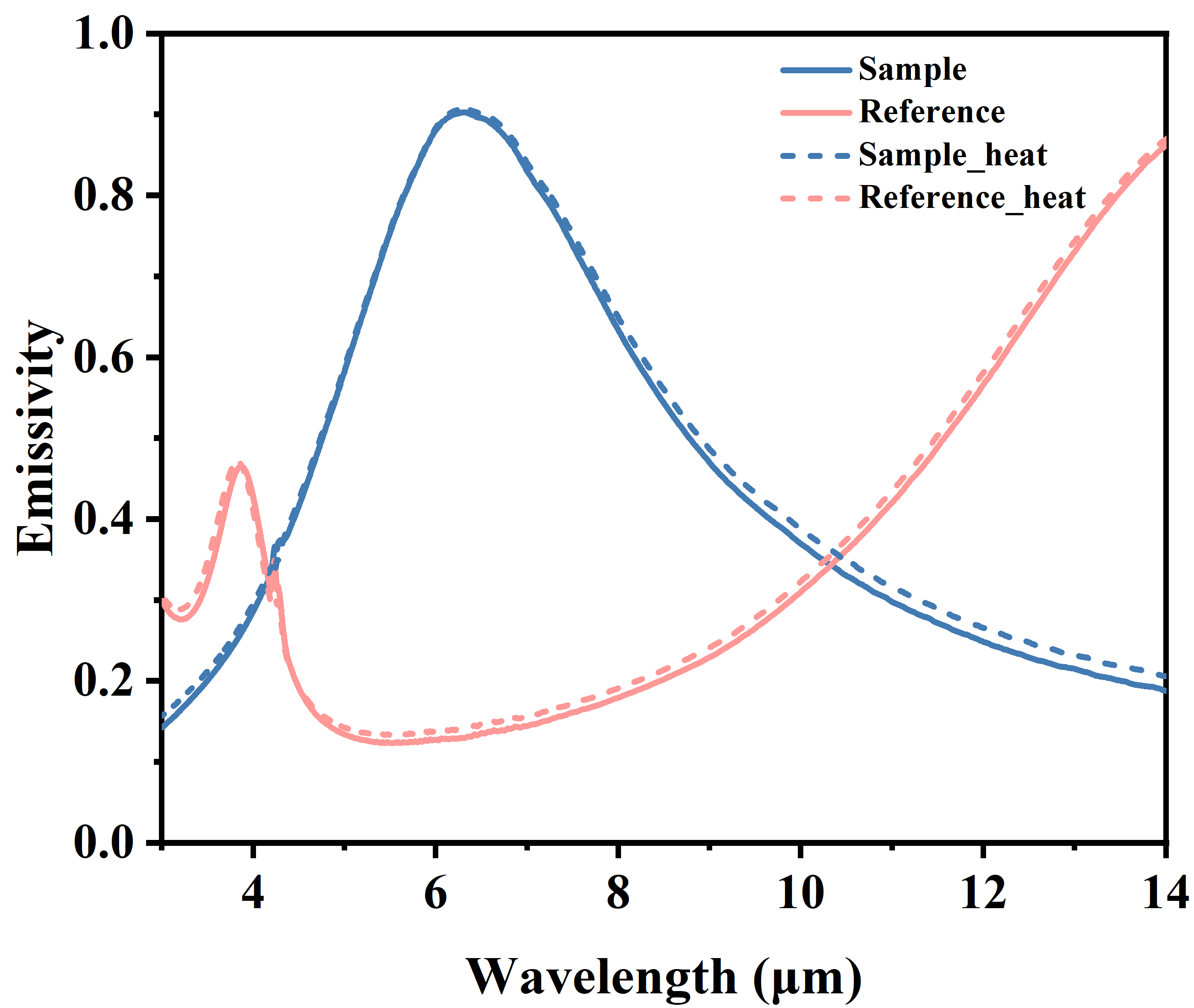


**Figure S4** Measured spectra of the sample and reference sample before and after heating at ~110 °C.

**Section S5 Numerical calculation of temperature difference between sample and reference sample.**

The numerical calculation of temperature difference is carried out by Finite Element Method (FEM). The sample and reference sample are both modeled as silicon cubes. The evaporated layers are ignored in heat transfer simulation since their ignored thermal capacity compared to Si substrate. The geometric parameters are both set to 1.5 cm (length),1.5 cm (width), and 0.5 mm (height), which is consistent to fabricated samples. To consider the thermal radiation, the emissivity of two top surfaces is set to the average emissivity of sample and reference sample at 110 °C calculated by measured spectra in 3 ~ 14 µm, which are 0.51 and 0.29, respectively. The bottom surfaces are set to heat flux boundary conditions. The heat flux is 1700 W/m^2^ in simulation, which is lower than the real heat power (2400 W/m^2^) of hot plate since the unperfect thermal contact of sample/reference sample with the hot plate. The results of stationary solution show the temperature of sample and reference sample are 104.9 °C and 112.1 °C, respectively. The temperature difference is 7.2 °C.

**Section S6 Emissivity measurement of tap, blackbody, water, and water with blue dye.**

The emission properties of tape and “blackbody” in Figure 6(b) are investigated. The reflectance and transmittance of tape with smooth surface are both measured (*E*=1 - *R* - *T*). The reflectance of “blackbody” is measured through integrated sphere, and the transmittance is supposed to be zero (*E*=1 - *R*). The simulated sea water is distilled water with several drops of blue dye. The reflectance of distilled water with and without blue dye is also measured by integrated sphere and the transmittance is also supposed to be zero because water is essentially opaque to infrared radiation longer than 3 µm[1, 2] (*E*=1 - *R*). Thus, the emissivity of tape and “blackbody” are calculated as shown in Figure S5 (a) and the emissivity of water with and without blue dye are as shown in Figure S5 (b). The tape and “blackbody” exhibit both high emissivity in 7.5 ~ 14 µm. Several drops of blue dye show no significant impact on the emissivity of water.


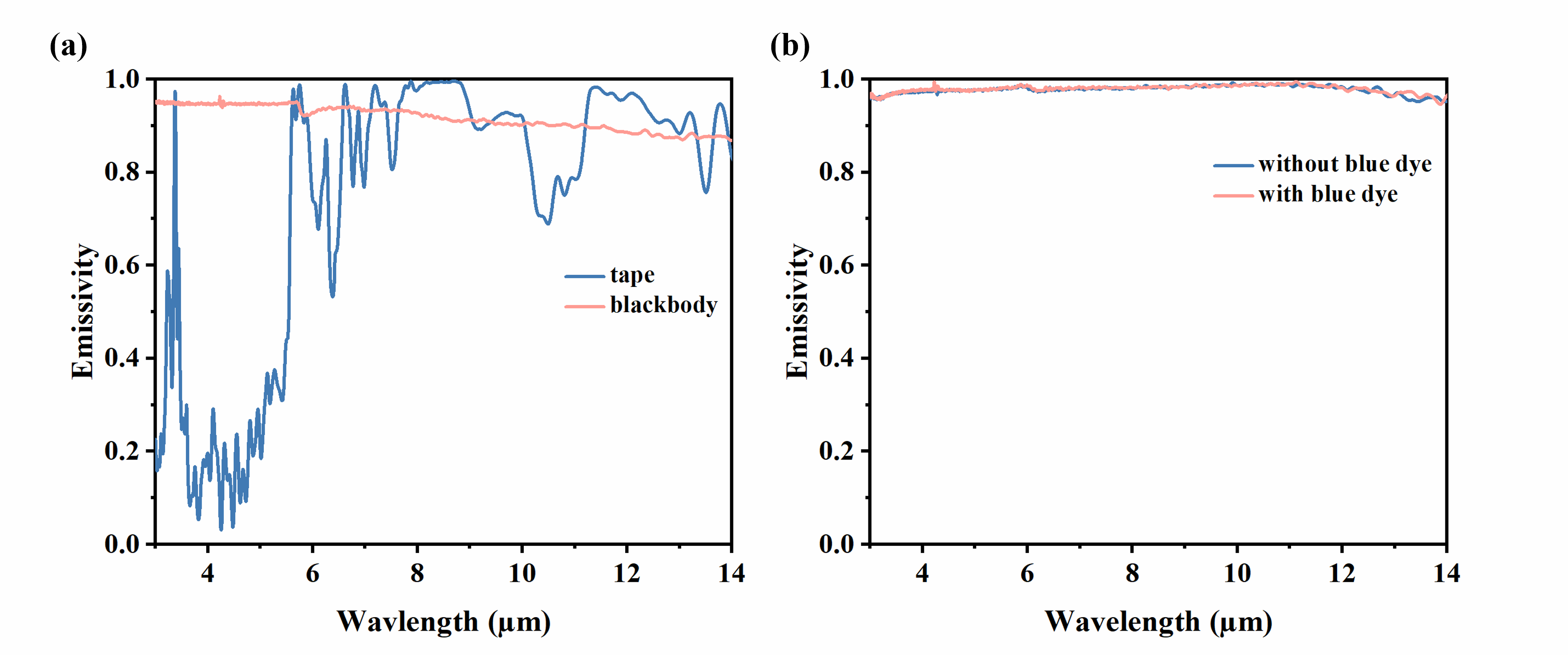


**Figure S5** (a) Measured emissivity of tape and near blackbody. (b) Measured emissivity of distilled water without and with blue dye.

**Reference**

[1] W. L. Wolfe and G. J. Zissis, *The Infrared Handbook*. Washington, DC: Environ-mental Research Institute of Michigan Ann Arbor InfraredInformation and Analysis Center, Office of Naval Research, 1978.

[2] B. Wozniak and J. Dera, *Light absorption in sea water*. New York: Springer Science+Business Media, 2007.
